# Remnant preservation provides good clinical outcomes after anterior cruciate ligament reconstruction

**DOI:** 10.1007/s00167-020-06406-6

**Published:** 2021-01-02

**Authors:** Hui Huang, Masashi Nagao, Hirofumi Nishio, Haruka Kaneko, Yoshitomo Saita, Yuji Takazawa, Hiroshi Ikeda, Kazuo Kaneko, Muneaki Ishijima

**Affiliations:** 1 Department of Orthopaedic Surgery, Juntendo University Graduate School of Medicine, 2-1-1 Hongo Bunkyo-Ku, Tokyo, 113-8421 Japan; 2 Medical Technology Innovation Center, Juntendo University, Tokyo, Japan; 3 Clinical Research and Trial Center, Juntendo University, Tokyo, Japan; 4 Graduate School of Health and Sports Science, Juntendo University, Chiba, Japan; 5 Sportology Center, Juntendo University Graduate School of Medicine, Tokyo, Japan

**Keywords:** Anterior cruciate ligament, Remnant preservation, Graft rupture, Return to play, Psychological effects

## Abstract

**Purpose:**

To evaluate the association of remnant preservation (RP) and non-RP (NRP) with patient-reported outcome measures and subsequent graft rupture at a minimum 2-year follow-up after anterior cruciate ligament (ACL) reconstruction.

**Methods:**

Patients in this retrospective study underwent primary isolated ACL reconstruction by the RP or NRP technique with a four- to five-strand hamstring tendon graft. Multivariate linear or logistic regression and Cox regression analyses were performed to compare the physical and psychological outcomes by the International Knee Documentation Committee subjective knee form (IKDC-SKF) and the Japanese Anterior Cruciate Ligament questionnaire 25 (JACL-25), respectively; satisfaction rate; and prognosticators of graft rupture.

**Results:**

In total, 120 patients (mean age, 30.6 ± 12.7 years; 54 RP, 66 NRP) with a mean follow-up of 3.2 ± 1.6 years were enrolled in this study. At the latest postoperative follow-up, the RP group showed a mean IKDC-SKF score of 92.3 ± 8.5 and mean JACL-25 score of 13.2 ± 11.2, while these scores in the NRP group were 86.4 ± 12.2 and 24.4 ± 19.5, respectively (*P* = 0.016 and 0.007, respectively). No significant differences were found in the return-to-sports rate (RP vs. NRP, 79.5% vs. 67.5%) or satisfaction rate (RP vs. NRP, 89.2% vs. 74.4%) (n.s.); however, a significant difference was found in the rate of return to the preinjury sports level (RP vs. NRP, 64.1% vs. 37.5%; *P* = 0.014). The graft rupture rate was significantly higher in the NRP than RP group (9/66 vs. 1/54; hazard ratio 9.29; 95% confidence interval 1.04–82.81). Younger age (≤ 18 years) was the other important risk factor for graft rupture (hazard ratio 8.67; 95% confidence interval 2.02–37.13).

**Conclusion:**

Patients who underwent ACL reconstruction with the RP technique obtained somewhat better physical and psychological results than those who underwent ACL reconstruction with the NRP technique. With respect to clinical relevance, patients treated with the RP technique may obtain better outcomes in terms of graft rupture and return to the preinjury sports level than those treated with the NRP technique, but with no differences in overall return to sports or satisfaction.

**Level of evidence:**

IV.

## Introduction

The surgical technique of anterior cruciate ligament (ACL) reconstruction with the remnant preservation (RP) technique has been widely described, and RP reportedly provides better physical function than the standard technique [18,21,34]. However, the benefits of RP remain debatable [24]. Several advantages of the RP technique are described in the current literature. For example, one study showed that the RP technique with single-bundle hamstring reconstruction resisted tibial tunnel enlargement (25.7% vs. 34.0%) [34], and another study showed that the RP technique with double-bundle (DB) hamstring reconstruction resulted in a smaller side-to-side difference than the standard technique (0.68 vs. 1.23 mm) [18]. However, one meta-analysis indicated that although better clinical outcomes were obtained with the RP than non-RP (NRP) technique (mean difference in Lysholm score, 2.20; side-to-side difference, − 0.71 mm), whether there is a definite advantage to using the RP technique instead of the NRP technique remains unclear because of the small differences in means [32]. Additionally, a prospective randomised controlled study showed no evident advantages in clinical outcomes in terms of stability, synovial coverage, or proprioception recovery [10]. Similar results have been reported in terms of knee stability and graft incorporation by clinical and magnetic resonance imaging evaluations [24].

At present, the benefits of the RP technique in terms of the psychological effects, return to the preinjury sports level, satisfaction rate, and incidence of ACL revision remain uncertain. Additionally, psychological factors, such as fear of re-injury, lack of confidence, and fear of graft failure, play an important role in a patient’s ability to return to play after ACL reconstruction surgery [2,6,17]. Consequently, it is necessary to confirm whether the RP technique can improve these outcomes.

Thus, in the present study, we compared the physical and psychological outcomes, satisfaction rate, return to play rate, and graft rupture rate after ACL between an RP group and NRP group with a minimum 2-year follow-up. The hypothesis of this study was that the RP group would have better outcomes in terms of both physical and psychological factors of patient-reported outcome measures (PROMs), satisfaction, the incidence of ACL revision, and the return to play rate. The findings of this study will be clinically relevant to the ongoing efforts of improving clinical outcomes of patients who undergo ACL reconstruction surgery.

## Materials and methods

This retrospective study (case series, evidence level IV) was approved by the institutional review board of our institution (Juntendo University Hospital; ID No. 19-240), and all patients provided consent to participate with no financial incentives.

A total of 360 consecutive patients underwent ACL reconstruction surgery from 1 January 2013 to 26 August 2017. The inclusion criteria for this study were an age of ≥ 16 years, no history of lower limb surgery, performance of primary isolated ACL reconstruction surgery with a semitendinosus autograft (patients who underwent concomitant reconstruction of the posterior cruciate ligament, medial collateral ligament, anterolateral ligament, or posterolateral corner were excluded), presence or absence of concomitant injury including meniscal tears or chondral lesions, and availability of PROMs with a minimum 2-year follow-up. Before surgical treatment, the patients underwent preoperative rehabilitation to restore their knee range of motion (ROM) if necessary [8]. The decision regarding whether to perform RP was based on the following criteria: attachment of remnant fibres to the femoral side, remnant with more than half volume, and coverage of synovial tissues with preservation of the blood supply. Thus, patients with a preserved remnant with less than half volume or without a blood supply were excluded from this study.

**Fig. 1:**
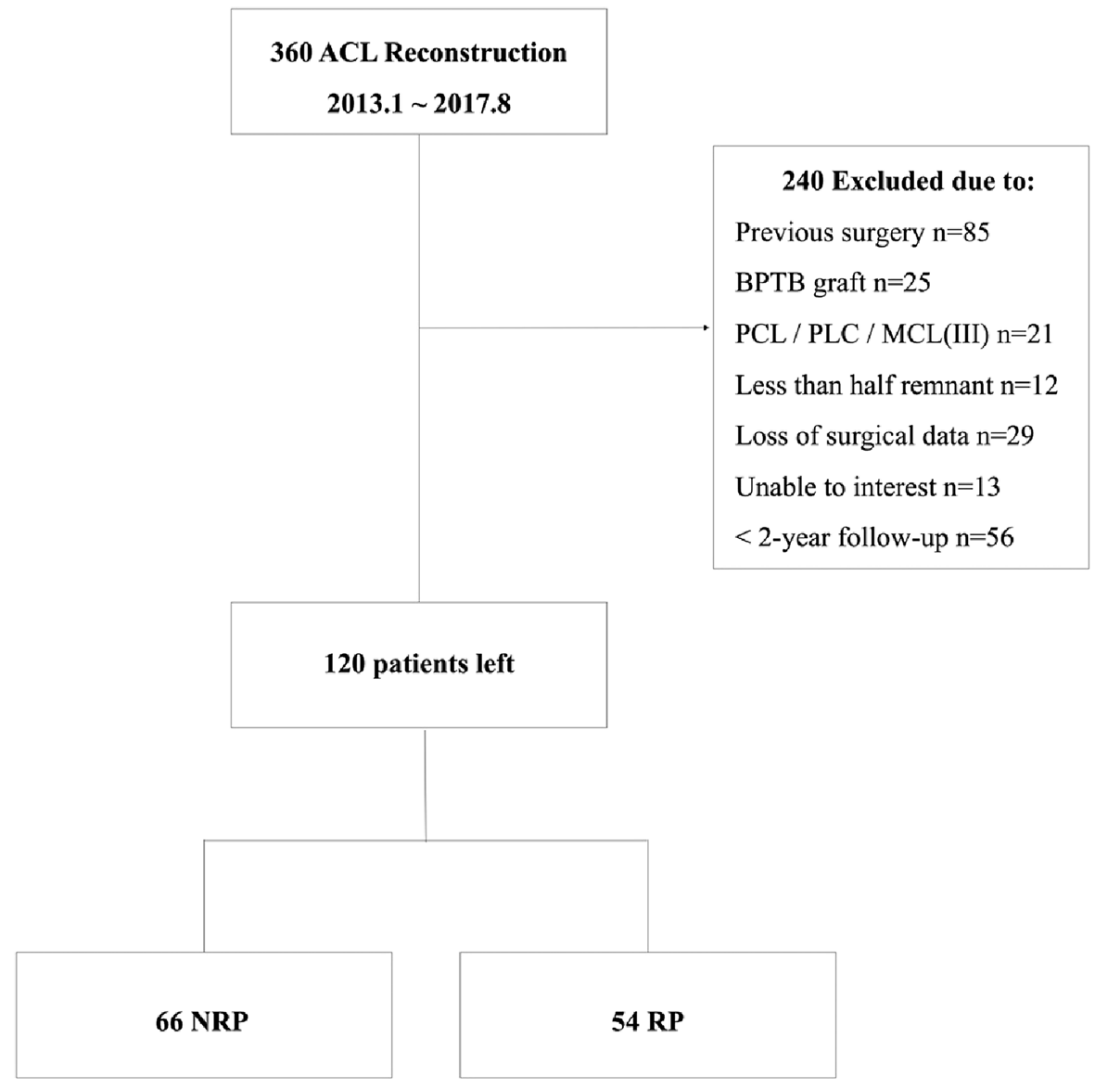
Flow chart. *ACL* anterior cruciate ligament, *BPTB* bone–patellar tendon–bone, *PCL* posterior cruciate ligament, *PLC* posterolateral corner, *MCL(III)* grade III medial collateral ligament injury, *NRP* non-remnant preservation, *RP* remnant preservation

During the study period, 120 patients (NRP, *n* = 66; RP, *n* = 54) were enrolled in this study (Fig. 1). The patients’ characteristics are presented in Table 1. The mean age of the cohort was 30.6 years, and 58 of 120 patients were male (48.3%). The mean duration of follow-up was 3.2 years. More than 55 patients underwent concomitant meniscal treatment with suture repair or meniscectomy, and 1 patient underwent treatment of chondral lesions with microfracture. There were no statistically significant differences between the RP and NRP groups in terms of the mean age, sex, affected side, duration from injury to surgery and follow-up, preinjury Tegner score, or graft size (n.s.). Statistically significant differences between the groups were only found in the body mass index (BMI) (*P* = 0.006) and meniscal treatment (*P* = 0.011).

**Table 1:** Patient demographics

	All patients (*n* = 120)	RP group (*n* = 54)	NRP group (*n* = 66)	*P* value
Age, mean ± SD, years	30.6 ± 12.7	31.9 ± 12.7	29.6 ± 12.7	n.s
Males/females, *n*	58/62	30/24	28/38	n.s
BMI, mean ± SD	23.6 ± 3.8	24.7 ± 4.1	22.7 ± 3.3	0.006*
Side—right, *n* (%)	63 (52.5)	33 (61.1)	30 (45.5)	n.s
Injury to surgery, *n* (%)				n.s
Within 10 weeks	30 (25)	18 (33.3)	12 (18.2)	
10 weeks to 6 months	47 (39.2)	20 (37.0)	27 (40.9)	
Over 6 months	43 (35.8)	16 (29.6)	27 (40.9)	
Follow-up, mean ± SD, years	3.2 ± 1.6	3.1 ± 1.5	3.3 ± 1.7	n.s
Pre-tegner score^+^, mean ± SD	7.2 ± 1.4	7.2 ± 1.5	7.2 ± 1.3	n.s
Graft size, mean ± SD, mm	9.4 ± 0.8	9.4 ± 0.8	9.4 ± 0.8	n.s
Meniscal treatment, *n* (%)				0.011*
Medial	30 (25.0)	9 (16.7)	21 (31.8)	
Lateral	33 (27.5)	10 (18.5)	23 (34.8)	
None	65 (54.2)	37 (68.5)	28 (42.4)	

*RP* remnant preservation, *NRP* non-remnant preservation, *SD* standard deviation, *BMI* body mass index, *n.s.* not significant

^+^Preinjury Tegner score; *P < 0.05

### Surgical technique with semitendinosus graft

**Fig. 2:**
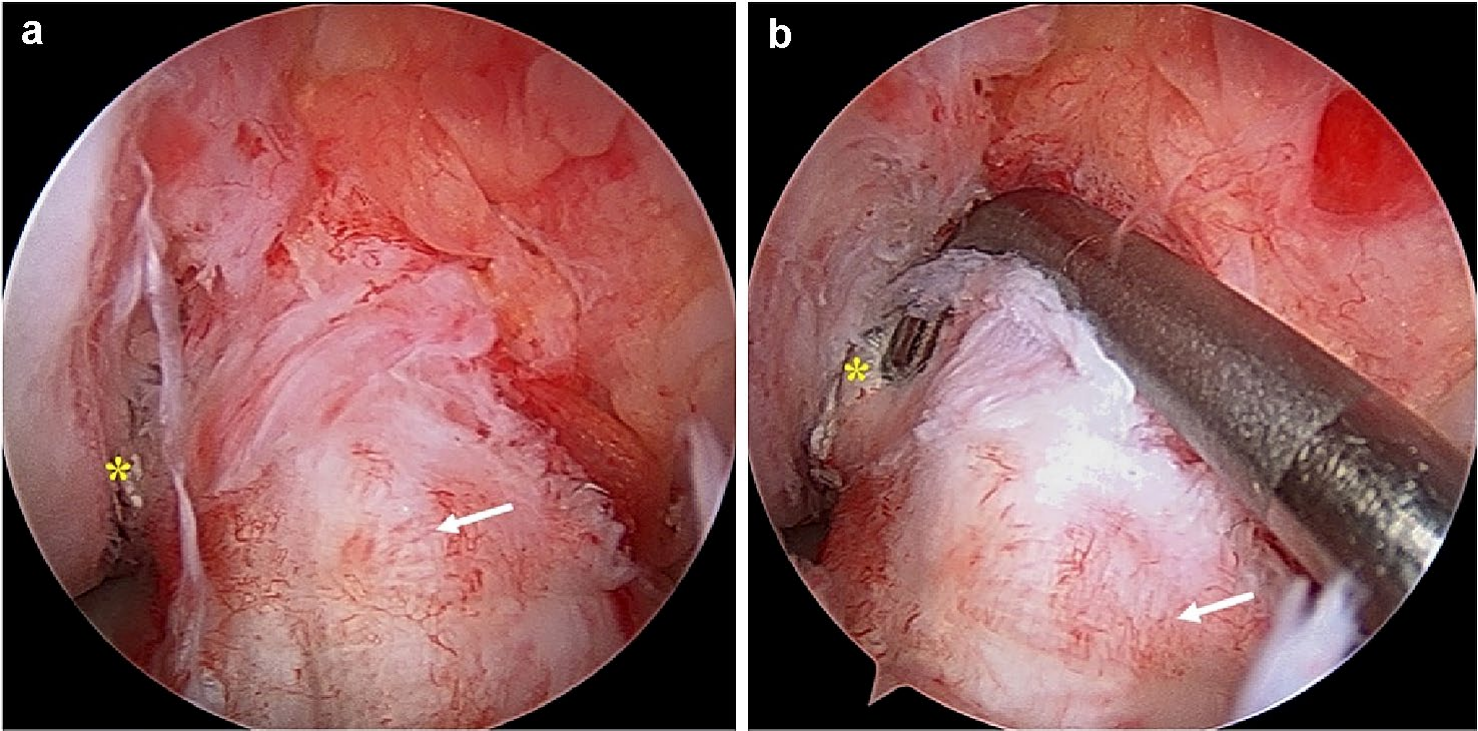
Illustration of anterior cruciate ligament reconstruction with remnant preservation (right knee). Reconstructed graft (asterisk) was covered by the preserved remnant with good synovial coverage (arrow)

Reconstruction surgery was performed by senior surgeons (HI, YT, YS, MN, and HK) with adequate experience in ACL reconstruction. Concomitant injuries, such as meniscal tears, chondral lesions, or loose bodies, were treated if indicated. The semitendinosus tendon was harvested by an open-ended tendon stripper to create a four- to five-strand single-bundle autograft. Before drilling the femoral side tunnel, unnecessary tissue was carefully cleaned to expose the lateral femoral footprint and preserve as much remnant as possible (Fig. 2). If the remnant condition was poor and did not meet the above-mentioned preservation criteria, the tissue was cleaned (NRP group). The preserved remnant was evaluated before skin closure after femora drilling. The femoral side bone tunnel was created with an inside-to-outside technique using the anteromedial portal technique [25] with a shallow depth, and a tibial side tunnel was then drilled with an outside-to-inside technique. The graft was passed from tibial to femoral side, fixed by the Telos Button (Ai-Medic, Tokyo, Japan) on the femoral side, and double-stapled on the tibial side.

### Postoperative management

All patients participated in a standardised postoperative rehabilitation programme. Full weight-bearing with knee brace-free, progressive ROM, and closed kinetic chain exercises were performed in the early rehabilitation period to restore full extension and quadriceps strength. Running and functional training was allowed from 3 to 4 months after surgery if the isokinetic strength and knee condition were acceptable. Non-contact pivot sports were allowed from 5 to 6 months, and contact pivot sports or preparation for return to play was allowed with a < 20% deficit in quadriceps strength, no pain, no restriction of knee function, and acceptable psychological confidence.

Physical examinations including ROM assessment, the Lachman test, and the pivot-shift test were performed preoperatively and postoperatively at an outpatient clinic by experienced orthopaedic surgeons. Because most of the patients were discharged from the outpatient clinic after the 1-year follow-up, long-term follow-up was performed by mailing a paper survey. The discharged patients were asked questions regarding any episodes of subsequent injury or surgery involving their ipsilateral or contralateral knee at other hospitals when they had returned to their preinjury sports level and the details of what had occurred if they had not returned to their preinjury sports level.

### Comparisons between RP and. NRP techniques

Subjective comparisons between the RP and NRP technique were performed using the International Knee Documentation Committee subjective knee form (IKDC-SKF) for physical outcomes (worst, 0; best, 100) and the Japanese Anterior Cruciate Ligament questionnaire 25 (JACL-25) [23] for psychological outcomes (best, 0; worst, 100). The JACL-25 was developed to assess fear of motion during daily activity and sports participation in patients with ACL injuries [13]. It contains 25 items with scores ranging from 0 to 100, and higher scores indicate worse psychological readiness. Additionally, the visual analogue scale for activities of daily living (VAS-ADL) (best, 0; worst, 100), the visual analogue scale for sports (VAS-Sports) (best, 0; worst, 100), the Tegner score (worst, 0; best, 10), and the Patient Acceptable Symptom State (Question: Taking into account all the activity you perform during your daily life, your level of pain, and your activity limitations and participation restrictions, do you consider the current state of your knee satisfactory? Answer: Yes or No) were used to evaluate activities of daily living, sports activities, level of sports participation, and postoperative satisfaction, respectively. The test–retest reliability for each PROM was assessed using the intra-class correlation coefficient with a two-way mixed model, with absolute agreement with a mean time interval of 2 weeks. The intra-class correlation coefficient for the IKDC-SKF, JACL-25, VAS-ADL, and VAS-Sports was 0.96, 0.98, 0.82, and 0.91, respectively. Finally, the incidence of ACL revision and the return to play rate (including the rate of return to the preinjury sports level) were also compared.

### Statistical analysis

All calculations were performed using RStudio for Mac version 1.1.423 (RStudio PBC, Boston, MA, USA), with statistical significance set at *P* < 0.05. The Shapiro–Wilk test was used to assess the normality of distributions. The baseline characteristics and the latest postoperative outcomes between the two groups were compared using the Mann–Whitney *U* test for outcomes and the chi-squared test or Fisher’s exact test for proportions. A multivariate linear regression model and a multivariate logistic model were used to perform an adjusted analysis during comparison of the postoperative outcomes. The Kaplan–Meier method was used to estimate the incidence of ACL graft revision, and the log-rank test was used to compare each group. Cox proportional-hazards regression models were used to investigate the association between the time of graft rupture before ACL revision and predictor variables. A post hoc test for the multivariable regression analysis was performed to determine whether the sample size of the study achieved sufficient statistical power to detect significant factors (G*Power 3.1; Heinrich-Heine-Universität Düsseldorf, Germany). The post hoc power analysis revealed that the sample size of 120 had sufficient statistical power of 0.82. Interactions between categorical variables were also tested.

## Results

**Fig. 3:**
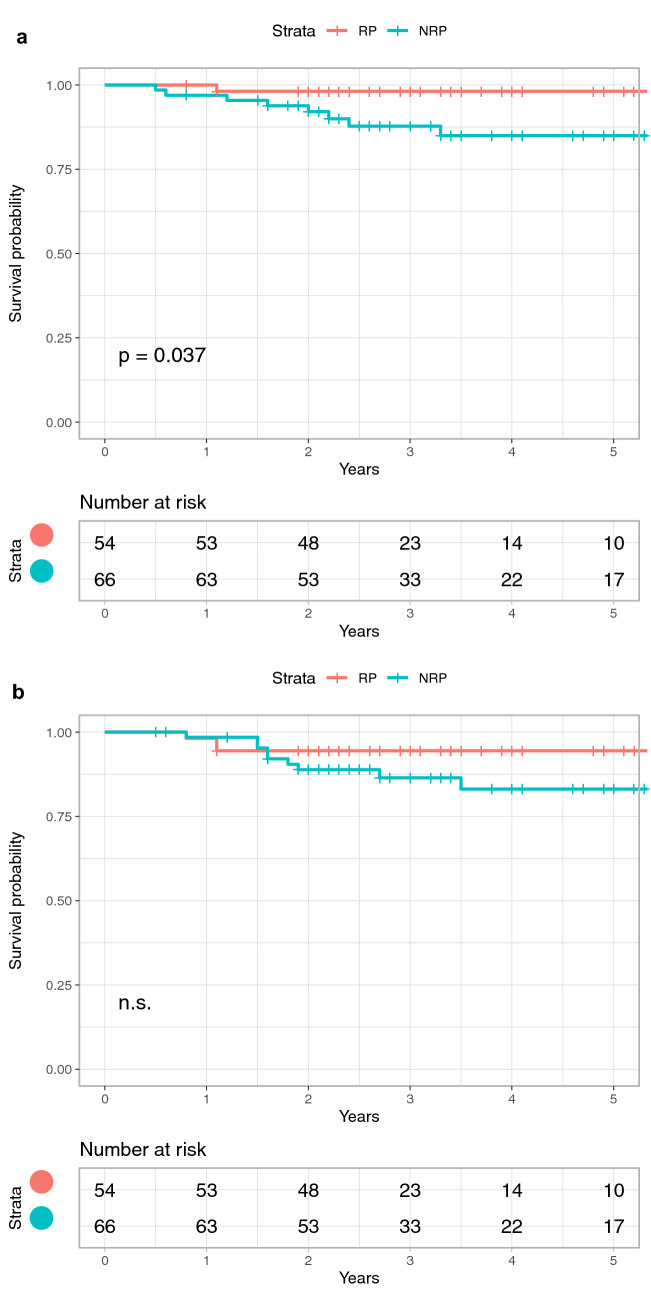
Kaplan–Meier plot of overall survival of **a** reconstructed graft and **b** contralateral anterior cruciate ligament tear. *RP* remnant preservation, *NRP* non-remnant preservation

The postoperative outcomes between the RP and NRP groups are shown in Table 2. The patients in the RP group had a better mean IKDC-SKF score [RP vs. NRP (mean ± standard deviation), 92.3 ± 8.5 vs. 86.4 ± 12.2; *P* = 0.016] and JACL-25 score (RP vs. NRP, 13.2 ± 11.2 vs. 24.4 ± 19.5; *P* = 0.007) than those in the NRP group, but there were no significant differences in the VAS-ADL score, VAS-Sports score, postoperative Tegner score, or rate of secondary meniscus surgery (n.s.). After controlling for the differences in demographics (BMI and meniscal treatment), significant differences were also found in both the IKDC-SKF score [coefficient, − 7.4; 95% confidence interval (95% CI), − 12.1 to − 1.9; *P* < 0.008) and JACL-25 score (coefficient, 9.9; 95% CI 2.0–17.8; *P* < 0.015). No significant differences were found in either the rate of return to sports or the satisfaction rate between the RP and NRP groups after adjustment for baseline differences (n.s.). However, the rate of return to the preinjury sports level was significantly higher in the RP than NRP group with an adjusted odds ratio of 3.01 (odds ratio, 3.01; 95% CI 1.09–9.29; *P* = 0.030). The overall proportion of graft rupture and contralateral ACL tears were 8.3% (10 of 120) and 10% (12 of 120), respectively. As shown in Fig. 3, the graft rupture rate in the RP and NRP groups was 1.9% (1 of 54) and 13.6% (9 of 66), respectively (*P* = 0.037), and the contralateral ACL tear rate in the RP and NRP groups was 5.6% (3 of 54) and 13.6% (9 of 66), respectively (n.s.). The mean times from surgery to graft rupture and contralateral ACL tear were 2.1 (95% CI 0.9–3.2) years and 1.7 (95% CI 1.2–2.1) years, respectively.

**Table 2:** Postoperative outcomes

	RP group	NRP group	*P* value
IKDC-SKF, mean ± SD	92.3 ± 8.5	86.4 ± 12.2	0.016*
JACL-25, mean ± SD	13.2 ± 11.2	24.4 ± 19.5	0.007*
VAS-ADL (0–100), mean ± SD	7.6 ± 9.5	9.9 ± 17.6	n.s
VAS-Sports (0–100), mean ± SD	17.6 ± 20.6	22.7 ± 20.9	n.s
Post-Tegner score^¶^, mean ± SD	6.6 ± 1.5	6.2 ± 1.5	n.s
PASS (%)	89.2	74.4	n.s
Return to play^§^ (%)	79.5	67.5	n.s
Return to preinjury level of sport (%)	64.1	37.5	0.014*
Secondary meniscus surgery	3	1	n.s

*RP* remnant preservation, *NRP* non-remnant preservation, *IKDC-SKF* International Knee Documentation Committee subjective knee form, *JACL-25* Japanese Anterior Cruciate Ligament questionnaire 25, *VAS-ADL* visual analogue scale for activities of daily living, *VAS-Sports* visual analogue scale for sports, *SD* standard deviation, *PASS* Patient Acceptable Symptom State, n.s. not significant

^¶^Postoperative Tegner score

^§^Returned to any sport

*P < 0.05

Based on the Cox proportional-hazards regression analysis, the graft rupture rate in the NRP group was 9.29 times higher than that in the RP group (95% CI 1.04–82.81) (Table 3). Predictive factors were also examined in the present study. Patients aged ≤ 18 years had a higher failure risk (graft rupture in younger patients: 6 of 17 in the NRP group and 1 of 9 in the RP group) than adult patients (hazard ratio 8.67; 95% CI 2.02–37.13) (Table 3). There were no significant differences in sex, BMI, or meniscal treatment (n.s.). Twenty-one patients (6 RP, 15 NRP) underwent secondary surgery after primary isolated ACL reconstruction. Of these patients, 6 of 10 (1 RP, 5 NRP) who developed graft rupture underwent revision surgery, 11 of 12 (2 RP, 9 NRP) underwent contralateral ACL reconstruction, and 4 (3 RP, 1 NRP) underwent secondary meniscal treatment (3 meniscectomies and 1 meniscectomy + meniscal suture). No patients underwent arthrosis for a cyclops lesion or cyclops syndrome.

**Table 3:** Predictive factors of graft rupture

	Adjusted HR	95% CI	*P* value
NRP vs. RP	9.29	1.04–82.81	0.046*
Age: ≤ 18 vs. > 18	8.67	2.02–37.13	0.004*
Gender: female vs. male	0.24	0.05–1.10	n.s
BMI: ≥ 25 vs. < 25	0.20	0.02–1.77	n.s
Meniscus: yes vs. no	0.53	0.13–2.16	n.s

*HR* hazard ratio, *CI* confidence interval, *RP* remnant preservation, *NRP* non-remnant preservation, *BMI* body mass index, *n.s.* not significant

*P < 0.05

## Discussion

The key finding of this study is that patients treated with the RP technique have significantly better physical functional outcomes and psychological outcomes than patients treated with the NRP technique. Additionally, reconstruction surgery with the RP technique showed a lower graft rupture rate and a higher rate of return to the preinjury sports level at the midterm follow-up than reconstruction with the NRP technique. Finally, both younger age and the NRP technique were associated with an increased risk of graft rupture.

Both physical and psychological readiness can reduce the risk of subsequent knee injuries [15,19,20]. To our knowledge, no studies to date have focused on the association between the RP or NRP technique and psychological factors in patients undergoing ACL reconstruction. Psychological factors including fear of re-injury, fear of graft rupture, or lack of confidence are among the most important factors that affect patients’ return to their preinjury sports activity after ACL reconstruction [2,29]. Ardern et al. [2] reported that less than half (40%) of patients returned to their preinjury sport level, with the most common reasons for not returning to sports being lack of trust in the knee (28%) and fear of sustaining a new injury (24%). In the present study, psychological readiness was also a main reason for patients not returning to their preinjury sports level. At follow-up, more than 59.1% of patients did not return to their preinjury sports because of fear of re-injury or graft rupture. Although no statistically significant differences were found in the rate of return to play between the RP and NRP groups in the crude analysis, the RP technique demonstrated a higher return to the preinjury sports level than did the NRP technique, with an adjusted odds ratio of 3.01.

With respect to the physical functional outcomes of the IKDC-SKF, previous randomised controlled trials have demonstrated no significant differences between the RP and NRP techniques after ACL reconstruction [3,7,28]. However, the surgical techniques in those studies were slightly different from ACL reconstruction with RP. The differences in the outcome measures might have been due to the different types of RP techniques that were utilised. For example, two of the three above-mentioned studies preserved the posterolateral bundle of the ACL and reconstructed an anteromedial graft using a hamstring graft. Additionally, one study involved the use of the DB hamstring tendon technique with minimal debridement. Furthermore, despite each of them being a randomised controlled trial, the method of blinding patients was either not reported or not used in these studies. Finally, the results might have also been affected by the study design, including a sample size of < 30 in each group, different mean ages, different periods from injury to surgery, and a follow-up duration of only 1 or 2 years. Thus, the risk of bias is unclear. Although the RP group had significantly better IKDC-SKF scores than the NRP group in the present study, the < 10.7-point difference in the IKDC-SKF score may not be clinically meaningful [12].

The condition of the preserved remnant may play an important role in the reconstructed graft remodelling and maturity. Kim et al. [14] presented the importance of synovial coverage in clinical outcomes and knee stability during ACL reconstruction with the RP technique. They demonstrated that RP with good synovial coverage had a positive effect on graft synovialisation and maintenance of graft integrity, but poor synovial coverage did not. This point has also been presented in DB ACL reconstruction with the RP technique [16]. In that study, the degree of initial graft coverage with RP significantly affected the postoperative knee stability after DB ACL reconstruction [16]. In a study involving a rabbit model, the blood flow was significantly higher in the RP than NRP group [33]. Moreover, the integration of the bone–tendon interface after reconstruction was also improved in the RP group. The blood supply of the native ACL originates primarily from the tibial and femoral insertions, which form a vascular plexus in the synovial membrane covering the ligament [4]. The preserved remnant with native vessels may provide the reconstructed graft a source of blood supply for revascularisation. It may accelerate graft remodelling and early graft restoration. Therefore, in the present study, the benefit of the RP technique with good synovial coverage and blood supply of the remnant likely contributed to the better outcomes with the RP than NRP technique.

Our study also demonstrated that the RP technique was associated with a lower graft rupture rate than the NRP technique in ACL reconstruction. The NRP technique was associated with a higher risk of graft rupture than the RP technique; however, because of the small number of graft ruptures and small sample size, the results may overstate the real association. Notably, this result is very similar to that obtained in a previous study [30]. The previous study also indicated that the mean side-to-side anterior stability as measured by the KT-2000 arthrometer was significantly lower in the RP than NRP group (1.0 ± 0.8 vs. 1.3 ± 1.0, respectively; *P* < 0.05). These results indirectly indicate that RP can provide better knee stability than NRP, as we discussed above. It is clinically important that good knee stability after ACL reconstruction can reduce subsequent knee damage, especially meniscal tears or degeneration and the progression of osteoarthritis [5]. Furthermore, younger age was also a clear prognosticator in the present study, although this is already known as one of the most important risk factors for revision [21,26].

In the present study, the baseline rate of meniscal injury was lower in the RP than NRP group, which may indirectly reflect the fact that injury was less severe in the RP than NRP group. However, after adjustment for differences in demographics (age, sex, and RP), no statistically significant differences were found in either the IKDC-SKF score or JACL-25 score (n.s.). Additionally, with respect to the association between concomitant meniscal injuries and knee instability, previous studies have indicated that concomitant meniscal injury is associated with increased knee rotatory laxity in patients with ACL insufficiency [9,11,22]. However, after the treatment of concomitant meniscal injury with meniscectomy or suture repair during ACL reconstruction, meniscal tear does not seem to be a risk factor for postoperative knee instability [1,9,31].

Twenty-one secondary surgeries were performed in this study. With the exception of revision surgeries and contralateral ACL reconstruction, meniscal treatment was the main reoperation in this cohort. Although a previous study showed that ACL reconstruction with the RP technique may increase the occurrence rate of cyclops lesions or cyclops syndrome [27], no patients developed this complication in the present study. Overall, at the minimum 2-year follow-up, ACL reconstruction with the RP technique provided good outcomes, although a further long-term study is needed.

This study has several limitations that should be acknowledged. The main limitation is its nonrandomised design. Although a randomised controlled trial can provide unbiased or minimally biased results, such a study may not be reasonable because of the difficulty of checking the remnant condition, including assessing the continuity of the remnant, blood flow, and synovial coverage by magnetic resonance imaging before surgery. The other main limitation of this study is its retrospective design and small sample size. This restricted the input of predictive variables during the multivariate linear or logistic analysis. Finally, the cohort also contained a potential risk of bias because of the wide range of ages and levels of sports participation.

Despite these limitations, this study suggests that ACL reconstruction with the RP technique is worthy of consideration because it can provide good clinical outcomes in terms of both graft failure and return to the preinjury sports level than the NRP technique if the preserved remnant is in good condition (attached to the femoral side, preservation of more than half volume, and coverage of synovial tissues with preservation of the blood supply). Moreover, ACL reconstruction with the RP technique may have more beneficial psychological effects than the NRP technique.

## Conclusion

The evidence in this study showed that patients who underwent ACL reconstruction with the RP technique obtained somewhat better outcomes in terms of both physical and psychological results than those who underwent ACL reconstruction with the NRP technique. With respect to clinical relevance, the results of this study suggest that patients treated with the RP technique may obtain better results in terms of graft rupture and return to the preinjury sports level than patients treated with the NRP technique, with no difference in the overall return to sports rate or satisfaction rate.
